# Feasibility and Diagnostic Accuracy of Saliva-Based SARS-CoV-2 Screening in Educational Settings and Children Aged <12 Years

**DOI:** 10.3390/diagnostics11101797

**Published:** 2021-09-29

**Authors:** Martin Hoch, Sebastian Vogel, Ute Eberle, Laura Kolberg, Valerie Gruenthaler, Volker Fingerle, Nikolaus Ackermann, Andreas Sing, Bernhard Liebl, Johannes Huebner, Simone Kuttiadan, Anita Rack-Hoch, Melanie Meyer-Buehn, Tilmann Schober, Ulrich von Both

**Affiliations:** 1 Department Task Force Infectious Diseases, Bavarian Health and Food Safety Authority, Lazarettstrasse 67, 80636 Munich, Germany; Martin.hoch@lgl.bayern.de (M.H.); Sebastian.vogel@lgl.bayern.de (S.V.); Valerie.gruenthaler@lgl.bayern.de (V.G.); Bernhard.liebl@lgl.bayern.de (B.L.); 2 Public Health Microbiology Unit, Bavarian Health and Food Safety Authority, Veterinaerstrasse 2, 85754 Oberschleissheim, Germany; Ute.eberle@lgl.bayern.de (U.E.); Volker.fingerle@lgl.bayern.de (V.F.); Nikolaus.ackermann@lgl.bayern.de (N.A.); Andreas.sing@lgl.bayern.de (A.S.); 3 Division of Pediatric Infectious Diseases, Dr. von Hauner Children’s Hospital, University Hospital, Ludwig-Maximilians-University, Lindwurmstrasse 4, 80337 Munich, Germany; Laura.kolberg@med.uni-muenchen.de (L.K.); Johannes.huebner@med.uni-muenchen.de (J.H.); Anita.rack@med.uni-muenchen.de (A.R.-H.); Melanie.meyer-buehn@med.uni-muenchen.de (M.M.-B.); Tilmann.schober@med.uni-muenchen.de (T.S.); 4 Public Health Department, City of Munich, Schwanthaler Strasse 69, 80336 Munich, Germany; Simone.kuttiadan@muenchen.de

**Keywords:** SARS-CoV-2, Salivette^®^, saliva sampling, primary school, childcare facilities

## Abstract

Children have been disproportionately affected during the COVID-19 pandemic. We aimed to assess a saliva-based algorithm for SARS-CoV-2 testing to be used in schools and childcare institutions under pandemic conditions. A weekly SARS-CoV-2 sentinel study in primary schools, kindergartens, and childcare facilities was conducted over a 12-week-period. In a sub-study covering 7 weeks, 1895 paired oropharyngeal and saliva samples were processed for SARS-CoV-2 rRT-PCR testing in both asymptomatic children (*n* = 1243) and staff (*n* = 652). Forty-nine additional concurrent swab and saliva samples were collected from SARS-CoV-2 infected patients (patient cohort). The Salivette^®^ system was used for saliva collection and assessed for feasibility and diagnostic performance. For children, a mean of 1.18 mL saliva could be obtained. Based on results from both cohorts, the Salivette^®^ testing algorithm demonstrated the specificity of 100% (95% CI 99.7–100) and sensitivity of 94.9% (95% CI 81.4–99.1) with oropharyngeal swabs as reference. Agreement between sampling systems was 100% for moderate to high viral load situations (defined as Ct-values <33 from oropharyngeal swabs). Comparative analysis of Ct-values derived from saliva vs. oropharyngeal swabs demonstrated a significant difference (mean 4.23; 95% CI 2.48–6.00). In conclusion, the Salivette^®^ system proved to be an easy-to-use, safe and feasible saliva collection method and a more pleasant alternative to oropharyngeal swabs for SARS-CoV-2 testing in children aged 3 years and above.

## 1. Introduction

Children, in particular the group of <12-year-olds, are known to be at reduced risk for suffering from COVID-19. However, they have been substantially affected by closures of schools, kindergartens, and childcare facilities in the ongoing pandemic [1,2]. Among many other reports, a recent commentary by Nagakumar and colleagues highlights the urgent need to develop strategies to safely operate schools while avoiding their closures [3]. Hence, scientists and public health leaders alike have been exploring options for coronavirus testing approaches in educational settings. Accumulating evidence points towards a rather low and stable transmission risk in educational institutions despite rising incidence rates in the population, as long as preventative hygiene measures and regular testing strategies are in place [4]. The ideal system to allow for large-scale test operations would be child-friendly, safe to perform, and ideally allow for self-sampling at home or at the appropriate childcare institution without the help of a medical professional. Various groups have explored different sampling methods from a range of clinical specimens, while naso-/oropharyngeal swabs are considered the gold standard [5,6,7,8]. Since no discernable differences in viral loads or detection rates had been demonstrated between naso- and oropharyngeal swabs, oropharyngeal swabs have been considered the primary choice for SARS-CoV-2 testing in children to minimize injuries on the nasopharyngeal route [6,9]. Numerous reports have described saliva sampling in adults as a reliable non-invasive method for SARS-CoV-2 testing with a sensitivity of >80% and a specificity of >95% compared to naso-/oropharyngeal swabs [10,11,12,13,14,15]. Only very few reports have addressed saliva sampling in pediatric cohorts [16]. Of note, two studies comparing naso-/oropharyngeal swabs and saliva samples in symptomatic children found an overall saliva sensitivity of 85.2% (up to 95.2% in patients with high viral load (≥10^4^ copies/mL) in nasopharyngal swabs) and 87.7%, respectively [17,18]. The Salivette^®^ system has been proposed as a device for collecting saliva for SARS-CoV-2 testing in adults [19,20,21,22]. However, feasibility and diagnostic performance of this system in children and for routine testing in educational settings have not been assessed.

Hence, the aim of our study was to establish and assess a practical, safe, and easy-to-use system for saliva collection in educational settings using the Salivette^®^ and subsequent rRT-PCR testing for SARS-CoV-2 for adult staff and children aged 3 years and above.

## 2. Materials and Methods

### 2.1. Study Setting and Cohorts

Between June and November 2020, we conducted a weekly SARS-CoV-2 sentinel study in primary schools, kindergartens, and childcare facilities in Munich following approval by the Ethics Committee of the Ludwig-Maximilians University Munich [4]. In a sub-study covering a 7-week period, a total of 1895 paired oropharyngeal and saliva samples were obtained from asymptomatic children and staff attending participating educational institutions. Written informed consent was obtained from all adult individuals and from all parents or legal guardians of children participating in this study.

**Figure 1:**
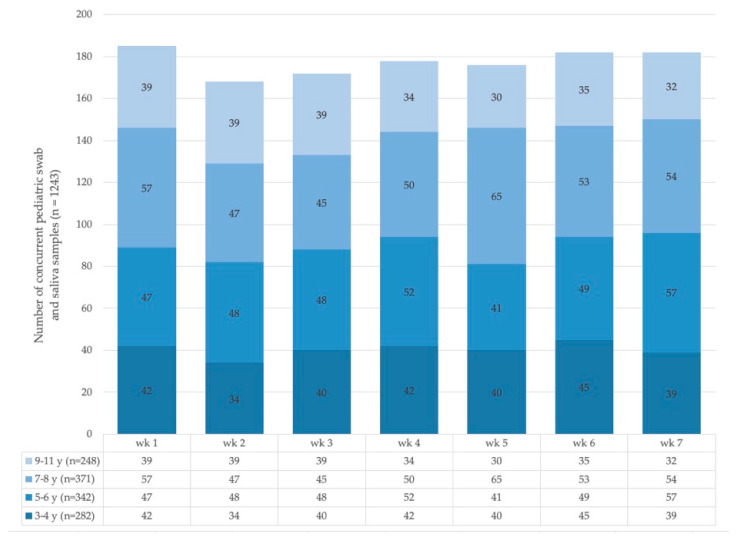
Pediatric sample pairs (oropharyngeal swab and Salivette^®^) were collected for SARS-CoV-2 rRT-PCR testing per study week. Colored bars illustrate stratification for individual age groups starting from the age of 3 years. y = years, wk = week.

The Salivette^®^ system was used for saliva sampling (SARSTEDT AG and Co KG, Nuembrecht, Germany; product number 51.1534). Over the 7-week period, we collected concurrent saliva and oropharyngeal swab sample pairs from children (*n* = 1243; Figure 1) and adult staff (*n* = 652)—sentinel cohort (SC, *n* = 1895). In parallel, 49 individuals, both adults and children known to be infected with SARS-CoV-2, were recruited and consented for prospectively paired Salivette^®^ and oropharyngeal swab sampling—patient cohort (PC, *n* = 49, Supplementary Table S1). PC participants were either recruited in the hospital inpatient setting or in collaboration with public health services by visiting quarantined individuals at home. To identify eligible individuals, the study team was notified of a positive PCR test for SARS-CoV-2 obtained during routine clinical sampling.

### 2.2. Salivette^®^ Sampling and Laboratory Processing

Supervised saliva sampling and swabbing were performed by medically trained study personnel in specifically assigned rooms on-site at the participating educational institutions. Samples were obtained after a minimum of 30 min since the last food and drink intake. Participants were asked to leave the Salivette^®^’s absorbent cotton pad in their mouth for a minimum of 2 min. Subsequently, each individual replaced the pad into the Salivette^®^ collection tube and closed it with the topper. Concurrent oropharyngeal swabs were taken immediately after saliva sampling. Following collection, samples were immediately transferred into the study laboratory. Salivette^®^ tubes were centrifuged for 5 min at 1600× *g* at room temperature to harvest saliva, following a protocol previously established in our children’s hospital’s routine diagnostic laboratory. A subset of samples was measured for saliva volume obtained by individual pipetting. All saliva specimens and swabs were processed using the ampliCube Coronavirus SARS-CoV-2 (Mikrogen, Neuried, Germany) on a Bio-Rad CFX96 Touch rRT-PCR Detection System (Bio-Rad Laboratories GmbH, Munich, Germany). Single gene results were retested with Xpert Xpress SARS-CoV-2 (Cepheid, Sunnyvale, CA, USA). For methodological comparison between swab and saliva sampling, we referred to semi-quantitative cycle threshold (Ct) values of corresponding SARS-CoV-2 gene locus.

### 2.3. Statistical Analysis

Statistical analysis was conducted using R-studio software, version 4.0.2.3 for chi-square test, Wilcoxon signed-rank test with continuity correction, and Bland–Altman graphical analysis [23].

## 3. Results

### 3.1. Feasibility of Salivette^®^ Sampling and Pre-Analytic Aspects

We were able to fully standardize saliva sampling by using the Salivette^®^. The risk for viral spreading was found to be negligible due to the closed collection system compared to more open systems (spitting, gargling) potentially producing aerosols. Furthermore, the tubes required minimal storage space and were compatible with standard centrifuges making large-scale laboratory processing very feasible. A subset of 875 individual samples (574 children, 301 staff) was subjugated to accurate measurements of saliva volume to explicitly address pre-analytic aspects. We found that for children across all age groups, a mean of 1.18 mL saliva could be obtained. For staff members of the participating institutions, a mean of 1.34 mL saliva could be collected (Table 1).

### 3.2. Salivette^®^ Diagnostic Performance

Of 1895 prospectively paired oropharyngeal swab and Salivette^®^ samples collected in the sentinel cohort (SC), 1893 showed a negative, and 2 samples yielded a positive result. Thus, as expected, the SC proved to be a low incidence cohort with only two positive pairs of samples detected in week 12 [4]. It, therefore, only allowed for accurate assessment of specificity of the Salivette^®^ method in relation to oropharyngeal swabs. As a consequence, we chose to establish an additional cohort (patient cohort, PC, *n* = 49) characterized by a high pre-test probability to evaluate the sensitivity of the Salivette^®^ testing method. The median age of this group was 46 years (range 3 to 87 years, male/female ratio 1.3; see Supplementary Table S1) and assessment of saliva volume per Salivette^®^ showed a mean of 1.73 mL (range: 0.75–2.75 mL). A total of eight individuals in the PC tested negative in both saliva and oropharyngeal swab samples. Thirty-seven individuals showed a positive test result from both sampling materials. Finally, two adult individuals demonstrated a discordant negative/positive and two additional adult individuals showed a discordant positive/negative result for Salivette^®^ and oropharyngeal swab samples, respectively. For negative saliva samples, Ct values from corresponding oropharyngeal swab samples were 33.17 and 33.72, while for negative oropharyngeal swab samples, Ct values from corresponding positive saliva samples read 37.49 and 37.68, respectively. No discordant results were seen for children aged <12 years.

Based on combined results from both cohorts, the Salivette^®^ testing method could be assigned a specificity of 100% (95% CI 99.7–100) and a sensitivity (percentage of positive agreement) of 94.9% (95% CI 81.4–99.1) in relation to oropharyngeal swabs (Table 2a,b).

### 3.3. Ct-Value Comparison

**Figure 2:**
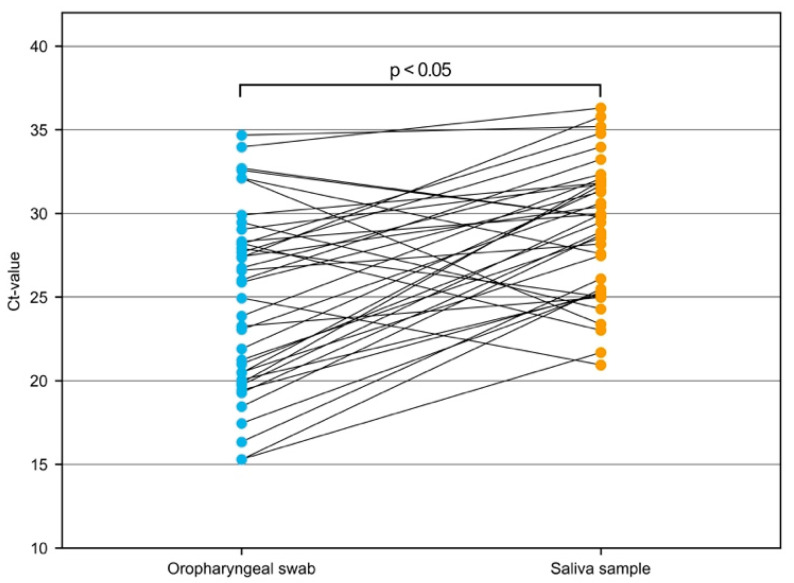
Comparison of cycle threshold (Ct) values of SARS-CoV-2 rRT-PCR corresponding gene loci from 39 Patient-matched saliva and oropharyngeal swab samples (SC: 2, PC: 37); *p*-value was calculated by Wilcoxon matched-pairs signed-rank test.

**Figure 3:**
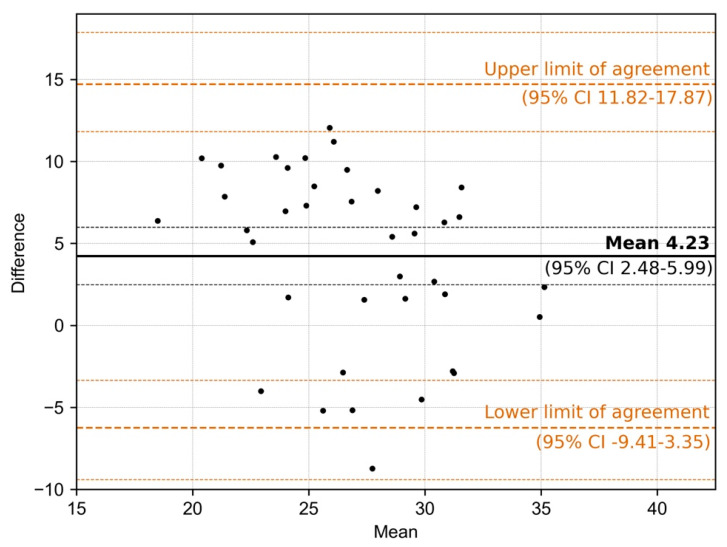
Bland-Altman graph displaying means and mean differences of Cycle threshold (Ct) values between 39 saliva and oropharyngeal swab sample pairs (SC: 2, PC: 37), including upper and lower limits of agreement.

To describe the effect of saliva sampling on Ct-value in comparison to oropharyngeal swabs, we assessed all Ct-values of individual sample pairs. Figure 2 and Figure 3 visualize patient-matched saliva and swab SARS-CoV-2 rRT-PCR Ct-values for respective 39 corresponding sample pairs (2 from SC and 37 from PC).

Wilcoxon signed-rank test with continuity correction showed a significant difference between Ct-value measurements derived from saliva vs. oropharyngeal swabs (*p*-value = 0.032). In addition, Bland–Altmann graphical comparison showed agreement between the two sampling methods with saliva-derived Ct-values being systematically higher than Ct-values derived from oropharyngeal swabs: mean difference 4.23 (95% CI 2.48–6.00), upper limit of agreement 14.85 (95% CI 17.87–11.82), and lower limit of agreement −6.38 (95% CI −9.41–−3.35) [23]. Ct-value data for all 39 sample pairs, including the age distribution of individuals tested, are listed in the supplementary material (Supplementary Table S2). Due to the limited number of available positive sample pairs from children aged <12 years, only three sample pairs of this age group were included in the Ct-value comparative analysis. The mean difference in Ct-values for these samples was 5.3 (range 0.52–8.42). In addition, we separately analyzed results of corresponding sample pairs for individuals with a moderate to high viral load (defined as Ct-values <33 from oropharyngeal swab samples) and were able to demonstrate that positive percent agreement between the two sampling systems was 100%.

## 4. Discussion

To our knowledge, this is the first large-scale feasibility study introducing the Salivette^®^ system in combination with rRT-PCR for SARS-CoV-2 testing for children (aged 3 years and above) and staff in educational settings. Thus far, only a few studies have reported the use of this testing algorithm in adults [19,24,25]. The Salivette^®^ system is an easy-to-use, safe, and feasible collection method licensed for supervised (professional use) saliva sampling in children aged 3 years and above. Its use for home sampling in 201 adults over a 2-week period was evaluated, comparing rRT-PCR results from saliva and oropharyngeal swabs [19]. Another recent study assessed Salivette^®^ as a standardized saliva collection device and compared SARS-CoV-2 positivity with paired nasopharyngeal swabs and saliva specimens in about 300 adults. The authors concluded that, when using nasopharyngeal swabs as a reference, Salivette^®^ samples showed a sensitivity and specificity of 82.9% and 91.4%, respectively. However, for samples containing less than 0.15 mL of saliva, the Salivette^®^ cotton roll was topped up with ultra-pure water and eluted again, introducing a relevant dilution effect prior to testing. This may explain the higher sensitivity demonstrated in our study since we did not have to top up any Salivette^®^-collected saliva sample and were thus always using neat saliva for SARS-CoV-2 testing.

We show that the mean difference in Ct-values between oropharyngeal swabs and saliva collected in the Salivette^®^ system was significant (4.23). Still, overall test specificity of 100% and sensitivity of 94.9% in relation to standard swabs demonstrated in this study were excellent. Of note, positive percent agreement between the two testing methods in individuals with a high to moderate viral load (Ct-values <33 from oropharyngeal swab samples) turned out to be 100%. This is of particular practical relevance since it proves that the Salivette^®^ system is not inferior to oropharyngeal swab sampling in the most relevant group of individuals [26]. However, only one rRT-PCR method was used in our study to assess both saliva and oropharyngeal swab specimens. Obtained Ct-Values were used for strict comparison between the two biological specimen types only. Some studies have demonstrated lower sensitivity and specificity of saliva testing methods, but this is most likely due to inadequate pre-sampling conditions and sample volumes [19]. Most reports do not explicitly address important pre-analytic aspects [8,13,27]. They frequently remain unclear about the volume of saliva collected and whether saliva samples were processed as neat material or diluted (buffer or normal saline) in the laboratory before rRT-PCR testing. To address pre-analytic consistency, we measured the volumes of harvested saliva. The results showed a consistent amount of saliva in adults and children. It has previously been demonstrated that supervised sampling, as in our study, yields better results than self-collection or oropharyngeal washing [15]. Finally, saliva test results are likely to be influenced by prior fluid or food intake, smoking, or other habits such as chewing gums. Melo Costa and colleagues recently assessed the concordance level between nasopharyngeal swab and Salivette^®^ samples in 319 paired samples from adults. They found that routine mouthwashes performed prior to obtaining saliva samples led to a substantial decrease in salivary viral loads, thus negatively impacting SARS-CoV-2 detection [22]. We ensured that saliva samples were not influenced by these factors. One may speculate that the best Salivette^®^ sampling window would be when sampling is integrated as an early-morning, pre-breakfast, and pre-toothbrushing routine procedure in the home setting.

One limitation of our study is based on its design of two independent cohorts. While our study clearly demonstrates both feasibility and highly reliable test performance of the Salivette^®^ system in the SC, the patient cohort for assessment of sensitivity was rather small. Comparison of Ct-values from concurrent sample pairs was only performed on 39 sample pairs. Since the SC proved to be a low incidence cohort with a low pre-test probability [4], we deliberately sought to establish the best cohort for assessment of sensitivity. Thus, our PC was characterized by a high pre-test probability. Hence it was only used to assess sensitivity and not designed to demonstrate specificity. In fact, specificity calculated from the PC would have shown a value of 80.0%, but with an extremely wide 95% confidence interval (CI) ranging from 44.2–96.5% (Table 2b). In contrast, the 95% CI for specificity derived from the SC was 99.7–100%, demonstrating a much more reliable result for specificity based on the SC. Assessing the Salivette^®^-saliva sensitivity in reference to oropharyngeal swab samples was based on a primarily non-pediatric cohort. In view of several reports comparing results of quantitative SARS-CoV-2 testing in both symptomatic and asymptomatic individuals of different age groups, it is reasonable to assume that there is no discernible difference in respiratory viral loads between children and adults [16,28,29]. In addition, since we were able to show that a sufficient (>1 mL) amount of saliva could be collected across all different age groups (Table 1), accurate and comparable processing of both adult and pediatric samples for SARS-CoV-2 rRT-PCR was ensured. Thus, sensitivity results obtained from the patient cohort may be skewed towards adults, and transferability to a pediatric setting may be limited. Still, the findings will certainly be of practical relevance for pediatric use. Of note, no discordant results were observed from children aged <12 years assessing concurrent pediatric saliva and oropharyngeal swab sample pairs. The four discordant results obtained from adult participants of the PC, a group of individuals all diagnosed with COVID-19 on a previous clinical rRT-PCR test result, are an important matter of debate. While the “saliva-negative/oropharyngeal swab-positive” pairs can easily be explained in view of the mean difference in Ct-values discussed above, we do not feel that the two “saliva positive/oropharyngeal swab-positive” pairs should be regarded as “false positive” since patients had a previously proven SARS-CoV-2 infection. In fact, we would interpret these positive results as “true positives” in view of consistent medical arguments. In a situation with SARS-CoV-2 infection subsiding in respective individuals (Ct-values 37.49 and 37.68), the Salivette^®^ samples may have been superior to oropharyngeal swabs in detecting the virus due to an increasingly patchy distribution of the virus in the oropharynx. This issue of negative swab results and SARS-CoV-2 detection in saliva samples as a correlate for the persistence of the virus in the body after oropharyngeal swab conversion has already been addressed and discussed by a number of groups [30,31]. Some authors even suggested to base evaluation of SARS-CoV-2 positivity on both oropharyngeal swab and subsequent saliva samples [31].

Our findings are further supported by a systematic review and meta-analysis comparing saliva and nasopharyngeal swabs for rRT-PCR testing for SARS-CoV-2 and demonstrating that both methods yield similar sensitivity and specificity across all 16 studies included in the analysis [10]. Other alternative non-oropharyngeal swab approaches have also been explored and may be practical for both adults and children. Whereas buccal swabs do not seem to be a reliable alternative option [32], Willeit and colleagues have reported promising results from gargling samples [33]. While this method may be feasible in adults and older children, it cannot be used in younger children. In addition, gargling involves external fluid or buffer, whereas the Salivette^®^ allows standardized collection of neat saliva as an undiluted clinical specimen. Furthermore, while Salivette^®^-based saliva collection, due to its closed system, is safe and not posing any risk for virus transmission to healthcare workers or friends and family nearby, gargling methods generate aerosols and are thus less suitable from an infection-control point of view. In view of recent evidence that SARS-CoV-2 also infects salivary glands and oral mucosa, saliva must be regarded as an optimal specimen of SARS-CoV-2 testing [34]. Furthermore, in situations where test capacities are limited, the Salivette^®^-collected individual saliva samples can easily be pooled in the laboratory to assess 5 or more single samples in one rRT-PCR run [35]. Thus, we fully agree with Anne Wyllie’s group, who recently concluded that standardized, inexpensive, and broadly implementable saliva-based methods could make frequent, comfortable testing for SARS-CoV-2 a reality for communities globally [36].

## 5. Conclusions

Our study assessing feasibility and diagnostic accuracy of the Salivette^®^ system for SARS-CoV-2 testing clearly demonstrated the specificity of 100% for a large group of >1200 children aged <12 years. Direct Ct-value comparison to calculate sensitivity (94.9%) was based on a smaller and primarily non-pediatric cohort and might thus be skewed towards adults. However, our results will still be of practical relevance for use in children. In view of the current pandemic situation with an increasingly rapid spread of coronavirus variants of concern and the B.1.617.2 (delta) variant, a Salivette^®^ based testing algorithm holds great potential for younger children, the largest yet unimmunized group of individuals, by ensuring safe operation of educational institutions.

## Figures and Tables

**Table 1:** Maximum, mean, and minimum amount of saliva collected using the Salivette^®^ system in children and staff (*n* = 875): Volume (mL).

Volume [mL]	3–4 Years (*n* = 145)	5–6 Years (*n* = 167)	7–8 Years (*n* = 170)	9–11 Years (*n* = 92)	All Children (*n* = 574)	Staff (*n* = 301)
Maximum	2.50	2.50	3.00	2.50		2.75
Mean	1.04	1.13	1.21	1.33	1.18	1.34
Minimum	0.20	0.20	0.20	0.25		0.20

**Table 2:** Patient-matched saliva (obtained using the Salivette^®^ collection system) and oropharyngeal swab data (rRT-PCR for SARS-CoV-2) for individuals tested for SARS-CoV-2, indicating specificity (a, sentinel cohort, SC) and sensitivity (b, patient cohort, PC).

		**Oropharyngeal Swab**	
(a) Sentinel cohort ^1^ (SC)		SARS-CoV-2 detected	SARS-CoV-2 not detected
saliva (Salivette^®^)	SARS-CoV-2 detected	2	0
	SARS-CoV-2 not detected	0	1893
	total	2	1893
Sensitivity: 100% (95% CI: 19.8–100%) **Specificity: 100% (95% CI: 99.7–100%)**			
		**Oropharyngeal Swab**	
(b) Patient cohort ^2^ (PC)		SARS-CoV-2 detected	SARS-CoV-2 not detected
saliva (Salivette^®^)	SARS-CoV-2 detected	37	2
	SARS-CoV-2 not detected	2	8
	total	39	10
Sensitivity: 94.9% (95% CI: 81.4–99.1%) **Specificity: 80% (95% CI: 44.2%–96.5%)**			

^1^ Due to its low incidence character SC did not allow calculating sensitivity. ^2^ Due to its high pre-test probability PC was not designed to assess specificity.

## Data Availability

The data presented in this study and not included in the supplementary material are available on request from the corresponding author. The data are not publicly available due to privacy and ethical reasons.

## Supplementary Materials

The following are available online at https://www.mdpi.com/article/10.3390/diagnostics11101797/s1, Table S1: Characteristics (age [years]; sex) of individuals recruited in the Patient Cohort (PC) used for sensitivity calculation (*n* = 49). Table S2: Age [years] sex and Ct-values of corresponding gene loci included in the head-to-head analysis (*n* = 39).
