# Supplementary material for: Feasibility and Diagnostic Accuracy of Saliva-Based SARS-CoV-2 Screening in Educational Settings and Children Aged <12 Years
Source: Diagnostics (Basel). 2021 Sep 29;11(10):1797. doi: 10.3390/diagnostics11101797 (PMC8534592; doi:10.3390/diagnostics11101797)
Supplement: Supplementary file 1 [file diagnostics-11-01797-s001.zip › diagnostics-1386902-supplementary-Table_S1_2021_09_28.pdf]

Supplementary Table (S1): Characteristics (age [years]; sex) of individuals recruited in the Patient Cohort (PC) used for sensitivity calculation (n = 49).

Age	Sex (Male, Female)	SAL	OPS
25	m	+	+
49	m	+	+
68	f	+	+
21	m	+	+
50	m	+	+
57	m	+	+
22	f	+	+
30	f	+	+
24	f	+	+
47	m	+	+
53	m	+	+
71	m	+	+
32	m	+	+
22	f	+	+
32	f	+	+
46	m	+	+
62	m	+	+
73	f	+	+
57	m	+	+
14	m	+	+
31	f	+	+
39	f	+	+
22	m	+	+
33	m	+	+
70	f	+	+
87	f	+	+
68	m	+	+
35	m	+	+
79	f	+	+
46	m	+	+
25	f	+	+
46	m	+	+
3	f	+	+
79	m	+	+
16	m	+	+
24	m	+	+
8	f	+	+
50	f	-	+
70	f	-	+
57	m	+	-
72	m	+	-
6	m	-	-
23	f	-	-
55	m	-	-
65	f	-	-
44	f	-	-
28	m	-	-
79	f	-	-
74	m	-	-

Abbreviations: SAL: Salivette® (Saliva); OPS: oropharyngeal swab.
