# Supplementary material for: Feasibility and Diagnostic Accuracy of Saliva-Based SARS-CoV-2 Screening in Educational Settings and Children Aged <12 Years
Source: Diagnostics (Basel). 2021 Sep 29;11(10):1797. doi: 10.3390/diagnostics11101797 (PMC8534592; doi:10.3390/diagnostics11101797)
Supplement: Supplementary file 1 [file diagnostics-11-01797-s001.zip › diagnostics-1386902-supplementary-Table_S2_2021_09_28.pdf]

Supplementary Table (S2): Age [years] sex and Ct-values of corresponding gene loci included in the head-to-head analysis (n = 39).

Age	Sex (Male, Female)	Ct-Value (SAL)	Ct-Value (OPS)
25	m	20.94	24.94
49	m	21.68	15.3
68	f	23.01	28.21
21	m	23.37	32.10
50	m	24.29	29.46
57	m	24.97	23.26
22	f	25.03	27.9
30	f	25.13	20.05
24	f	25.23	19.43
47	m	25.48	15.29
53	m	26.09	16.34
6	m	27.48	20.51
71	m	27.59	32.11
32	m	28.17	26.6
22	f	28.54	21.24
32	f	28.72	18.45
46	m	28.89	19.28
35	f	29.47	20.99
62	m	29.79	32.58
73	f	29.79	32.7
57	m	29.95	19.74
14	m	29.96	28.33
31	f	30.41	27.42
39	f	30.62	23.06
22	m	31.29	25.88
33	m	31.39	21.9
70	f	31.67	20.47
87	f	31.73	29.05
68	m	31.81	29.9
35	m	31.92	19.87
79	f	32.07	23.86
46	m	32.34	26.74
25	f	33.97	27.68
46	m	34.78	28.17
3	f	35.19	34.67
79	m	36.3	33.97
16	m	25.30	17.45
24	m	33.23	26.01
8	f	35.78	27.36

Abbreviations: SAL: Salivette® (Saliva); OPS: oropharyngeal swab.
